# Development of a Membrane Module Prototype for Oxygen Separation in Industrial Applications

**DOI:** 10.3390/membranes12020167

**Published:** 2022-01-30

**Authors:** Francesca Drago, Paolo Fedeli, Angelo Cavaliere, Andrea Cammi, Stefano Passoni, Riccardo Mereu, Stefano De La Pierre, Federico Smeacetto, Monica Ferraris

**Affiliations:** 1 Materials and Generation Technologies Department (TGM), Ricerca sul Sistema Energetico S.p.A.—RSE, 20134 Milan, Italy; paolo.fedeli@rse-web.it (P.F.); angelo.cavaliere@rse-web.it (A.C.); andrea.cammi@rse-web.it (A.C.); 2 Energy Department, Politecnico di Milano, 20156 Milan, Italy; stefano.passoni@polimi.it (S.P.); riccardo.mereu@polimi.it (R.M.); 3 Department of Applied Science and Technology (DISAT), Politecnico di Torino, 10129 Turin, Italy; stefano.delapierre@polito.it (S.D.L.P.); federico.smeacetto@polito.it (F.S.); monica.ferraris@polito.it (M.F.)

**Keywords:** oxygen transport membrane, LSCF, perovskite, membrane module, CFD model, metal-ceramic joining

## Abstract

The integration of oxygen transport membranes in industrial processes can lead to energy and economic advantages, but proof of concept membrane modules are highly necessary to demonstrate the feasibility of this technology. In this work, we describe the development of a lab-scale module through a comprehensive study that takes into consideration all the relevant technological aspects to achieve a prototype ready to be operated in industrial environment. We employed scalable techniques to manufacture planar La_0.6_Sr_0.4_Co_0.2_Fe_0.8_O_3-δ_ membrane components suitable for the application in both 3- and 4-end mode, designed with a geometry that guarantees a failure probability under real operating conditions as low as 2.2 × 10^−6^. The asymmetric membranes that act as separation layers showed a permeation of approx. 3 NmL/min/cm^2^ at 900 °C in air/He gradient, with a remarkable stability up to 720 h, and we used permeation results to develop a CFD model that describes the influence of the working conditions on the module performance. The housing of the membrane component is an Inconel 625 case joined to the membrane component by means of a custom-developed glass–ceramic sealant that exhibited a remarkable thermo-chemical compatibility both with metal and ceramic, despite the appearance of chemical strain in LSCF at high temperature. The multi-disciplinary approach followed in this work is suitable to be adapted to other module concepts based on membrane components with different dimensions, layouts or materials.

## 1. Introduction

In order to mitigate climate changes and activate the necessary energy transition, the European Union defined challenging targets in terms of reduction of greenhouse gas emissions, which must be achieved by 2050, with important implications on the energy system. In this context, the improvement of energy efficiency is a priority, and the entire industrial world plays an important role in such a transformation. In particular, energy-intensive processes need to be more energy efficient [1].

According with the European directives, Italy’s National Energy Strategy aims at reducing the final energy consumption, increasing renewable sources and contributing to the decarbonization of the electric system [2].

In order to promote the spread of energy efficiency in the industrial sector, the research must contribute to the development of new technologies and must overcome the technical barriers that can hamper industrial growth.

Based on the experience gained in the FP7 EU project GREEN-CC [3,4], we oriented our efforts toward oxygen transport membranes (OTMs), with the aim of developing a planar membrane module for oxygen separation at high temperature for integration in industrial processes. Such a technology has the potential to perform oxygen separation from air more efficiently than standard technologies [5,6,7,8,9,10,11].

The research of new possible applications of membrane modules in industrial sectors is essential to evaluate the cost-effectiveness and to increase the attractiveness of this technology, and to identify the operating conditions for the membrane material development and characterization. An example of OTMs integration was already proposed in studies [12,13]. At the same time, the technical feasibility of such innovative solution needs to be demonstrated; therefore, functional testing in an industrial environment is a key step for the validation of a new system, which requires the scale-up of ceramic components and the development of a proof-of-concept prototype. Different membrane module concepts were investigated by several research groups; for example, a tubular design based on extruded BSCF membranes was selected both by Fraunhofer Institute of Ceramic Technologies and Systems (IKTS) to develop an oxygen production unit with high energy efficiency [14], by RWTH Aachen University in the framework of the OXYCOAL–AC research project for the development of a module with an oxygen production of about 0.5 ton/day [8], and by the research group of the EU project HETMOC to design and test a module with 7 tubes that were 70 cm long [15]. A planar membrane module configuration was suggested by Air Products in the United States for the construction of a 5 ton/day oxygen separation prototype which can operate for over 1000 days [16]. All the mentioned concepts operate in a three-end mode configuration and thus in the absence of sweep gas. Other studies have dealt with planar systems operating in a sweep gas mode. In the frame of the FP7 European Union project, GREEN-CC, Schulze-Küppers et al. developed the manufacturing of OTMs components with dimensions of 70 × 100 mm^2^ suitable to be integrated in a module for oxy-combustion applications [3]. Moreover, Escolástico et al. reported the assembling and testing of a compact metallic reactor, which can operate either in co- or counter-current mode, with a membrane diameter of 13 mm [17].

All these works indicate that the planar configuration allows for a high degree of flexibility in the operation and integration of the membrane technology, both with and without sweep gas. At the same time, they point out that the technology readiness level of planar membrane systems operating in four-end mode still needs to be improved.

The membrane preparation based on the use of commercial powders, which allows for large-scale production, was already demonstrated [4], and the scalability of the manufacturing process was already reported in the literature for several ceramic membrane materials [3,18,19,20]. Nevertheless, some technical barriers in the membrane module construction are still present, making difficult its development and reliable utilization for oxygen production in industrial applications. Our main purpose was to design and develop a membrane module prototype with a channeled structure able to withstand the real industrial operating conditions, by taking into account each aspect of manufacturing, characterization and assembling of the membrane-based system in order to overcome the difficulties encountered in each step, to demonstrate the feasibility of such innovative technology, and to guarantee the future market penetration.

In this work, an overview of our recent activities on OTMs is reported, highlighting the multidisciplinary aspect of the research program and focusing the attention on the experimental activities finalized in the membrane module development, such as:

- Membrane manufacturing and scale up;
- Membrane characterization and testing;
- Membrane module design and joining.

## 2. Materials and Methods

### 2.1. Manufacturing and Scale Up

Commercially available materials were used for the manufacturing of OTMs components in order to demonstrate and improve the scalability of the process.

The different layers of the membrane components were manufactured by tape casting. LSCF powders, with nominal composition La_0.6_Sr_0.4_Co_0.2_Fe_0.8_O_3-δ_, were purchased from Praxair Inc. (Danbury, CT, USA) and fully characterized, as described in [4]. Slurries suitable for tape casting were prepared by dispersing the powders in a mixture of solvents and by adding appropriate amounts of organic binder and plasticizers. Two different slurries were prepared for casting thin dense layers and thick, porous supports, respectively, following the procedure schematized in Figure S1. Further details on slurry composition and preparation route can be found in [4].

A tape casting machine Mistler TTC-1200 (Richard E. Mistler Inc., Morrisville, PA, USA) was used to prepare two different kinds of green tapes:

- Asymmetric LSCF tapes obtained by sequentially casting the thin dense membrane layer and, after drying, the porous support directly on top of it (see Figure S2).
- Single-layered LSCF tapes to be used as porous interlayers in the final membrane component, obtained by casting the same slurry used for the support layer.

All layers were casted on a silicone-coated Mylar carrier, using a casting speed of 1 cm/s. Other relevant process parameters are summarized in Table 1.

After drying, the tapes were cut into rectangular pieces with dimensions of 110 mm × 80 mm, and the interlayer samples (obtained from tape ii) were cut to form a channeled structure. Punching out of the flow channels was performed by a hydraulic machine MTS 312.31 (MTS Systems Corp., Eden Prairie, MN, USA) equipped with custom-made metal plates, using a proper cutting tool (Karl Marbach GmbH, Heilbronn, Germany).

Membrane components ready to be integrated in the final module were fabricated by assembling two “half-components”, each composed of an asymmetric membrane laminated with a channeled interlayer. Lamination was performed by warm pressing the two layers using the same machine employed for punching, equipped with a climatic chamber MTS 651.12C. Before any lamination process, the temperature of the metal plates was set to 70 °C, and the plates were allowed to reach the thermal equilibrium.

Half-components were debinded and sintered in a Nabertherm N150/H DB200 furnace (Nabertherm GmbH, Lilienthal, Germany). Due to the high organic content in the green tapes, a slow debinding process was required to preserve the integrity of the samples, with heating rates not exceeding 0.2 °C/min in the first part of the treatment (RT ≤ T ≤ 500 °C). The sintering step was carried out at 1300 °C for 5 h. The duration of the complete thermal treatment was ~140 h. Samples showed a shrinkage of ~25% after sintering.

Sintered half-components were coupled by joining the two interlayer sides with a garnishing paste, consisting of 76 wt% LSCF powders, 22 wt% dibasic ester (Merck KGaA, Darmstadt, Germany) and 2 wt% Nuosperse FX9086 dispersing agent (Elementis Specialties, Inc., London, UK) mixed by using a planetary homogenizer “Thinky mixer” ARV-310 (Thinky Corp., Tokyo, Japan). The paste was rolled on both the interlayer faces, and then the two half-components were combined, paying attention to the proper alignment of the flow channels. Subsequently, the garnishing paste was sintered at 1200 °C for 5 h. During sintering, a load of ~400 g was placed on top to flatten the component and to improve the resistance of the joining.

Afterward, the component was ground to the final dimensions of 60 mm × 60 mm, and the lateral porous surfaces were sealed by brushing a thin layer of the garnishing paste, followed by sintering at 1200 °C for 5 h.

### 2.2. Characterization Techniques and Testing

Microstructure of sintered samples was characterized by scanning electron microscopy (SEM) using a Tescan Mira 3 field emission microscope (FESEM) operated at 20 kV. Analyses were performed in cross-section on samples embedded in resin (Epofix, Struers ApS, Ballerup, Denmark) and were polished and coated with a conductive graphite layer. Morphological parameters, including layer thickness, porosity and pore opening diameter, were estimated by quantitative image analysis on 10 different fields, using the software Nikon NIS-Elements. Open porosity was also calculated according to the Archimedes’ method [21].

X-ray diffraction (XRD) post-exercise characterization was conducted on membranes using a Bruker D2 diffractometer (Bruker GmbH, Karlsruhe, Germany) equipped with a Cu tube operated at 30 kV and 10 mA. Measurements were performed in Bragg–Brentano geometry using the Cu K_α_ radiation (λ = 1.5406 Å).

The thermo-mechanical behavior was investigated by differential dilatometry using a Netzsch 402 ED push-rod dilatometer (Netzsch–Gerätebau GmbH, Selb, Germany). Measurements were performed in the temperature range 50–1000 °C (heating/cooling rate of 5 °C/min) on prismatic samples with dimensions of 9 mm × 6 mm × 1 mm, cut from uniaxially dry-pressed disks sintered at 1300 °C for 5 h. Characterization was carried out in static air and argon (Ar).

Membrane performance was verified on asymmetric membrane samples with a diameter of 15 mm, a dense membrane thickness of about 10–20 µm and a porous layer of about 0.7 mm. First, the membrane density was verified by determining the helium permeance as a function of pressure at room temperature. Then, permeation tests were carried out in a quartz permeation cell similar to the one described in [22], where the membrane was placed between two pure gold O-rings in order to ensure a proper sealing between the ceramic sample and the cell at high temperature (approx. 1050 °C). The configuration of the experimental set up is reported in [13]. Tests were performed in the temperature range 1050–700 °C, at atmospheric pressure, by feeding synthetic air or pure oxygen as process gas on the membrane layer, with a constant flow rate of 250 or 200 Ncm^3^/min, respectively, while pure helium was fed as sweep gas on the support side of the sample, with a flow rate ranging between 100 and 300 Ncm^3^/min. The temperature was monitored by a Pt/Pt-Rh thermocouple placed next to the membrane sample and an additional thermocouple was placed on the external side of the permeation cell, in order to verify the substantially uniform temperature distribution. A micro-Gas Chromatographer (microGC) Varian CP4900 was used to measure the helium and the oxygen concentration in the permeate stream and to quantify the oxygen permeation. Moreover, the nitrogen concentration in the permeate was continuously monitored to estimate the membrane leakage, according to the procedure reported by Schlehuber [23].

At 1050 °C, the oxygen leakage of the tested samples was lower than 2%, while the maximum experimental error estimated on the oxygen flux was about 5%.

**Figure 1:**
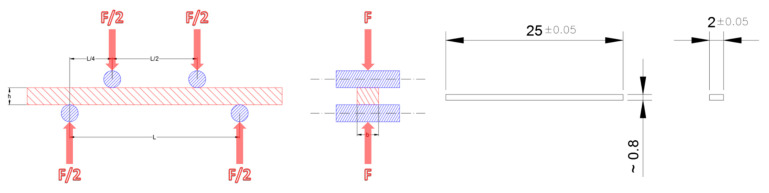
Symmetric four-point bending test configuration and test specimen geometry.

A mechanical characterization of the material was conducted by means of symmetric four-point bending tests at room and elevated (950 °C) temperature. Test configuration and specimen dimensions are reported in Figure 1.

Room temperature bending tests were performed using a custom-made device installed on an Instron 4507 electromechanical frame (Instron, Norwood, MA, USA), according to [24,25], in order to determine the flexural strength σ_f_ and Young’s modulus *E*. The test set up is reported in Figure S3a.

High-temperature tests at 950 °C were then performed heating the specimens by means of a resistance furnace (Severn Thermal Solutions, Dursley, UK) and subsequentially bending the samples by a custom made four-point SiC fixture installed on the Instron frame, as shown in Figure S3b. Test were carried out according to [25,26], in order to determine the flexural strength σ_f_ and Young’s modulus *E* at working conditions of the membrane.

Data resulting from mechanical characterization were treated according to the requirements of the international standards concerning the characterization of ceramic materials [24,26] and assuming that the ultimate tensile stress of the tested samples follows a two-parameter Weibull distribution.

### 2.3. Module Design and Joining

#### 2.3.1. Mechanical Design of Ceramic Component and Metallic Case

As the starting point of the design stage, we assumed for the scale test element (that is, a “ceramic component”) a parallelepiped with dimensions 60 mm × 60 mm × 5 mm. A channeled configuration was proposed in order to guarantee the operation both in 3-end mode and 4-end mode configuration (i.e., in the absence or presence of sweep gas, respectively), to ensure several integration possibilities, as reported in the membrane integration studies [12,13] and to increase the market appeal for such technology.

During operation, air flows along upper and lower faces of the element, while the permeated oxygen is gathered by a sweep-gas flowing inside internal channels. The number and size of these channels, as well as the width of the stability elements—that is, the distance between adjacent channels—were designed to obtain a reasonable speed of gas according with its desired flow rate and to satisfy the need of structural strength of the device.

FEM analysis, carried out by using the commercial software Comsol Multiphysics^®^, was performed to evaluate the stress distribution in a ceramic component that was preliminarily considered subjected only to different internal and external gas pressure, neglecting effects due to interaction with the module case. The failure probability of the module was computed, combining the failure probability in every cell of the model, and in turn evaluated through local stress conditions and data representative of the material characteristics, expressed in terms of its Weibull parameters.

For the membrane component case, we selected Inconel 625 as the metallic material, due to its mechanical characteristics and resistance to high temperature corrosion, but also to its thermal expansion coefficient, well matching that of LSCF.

#### 2.3.2. CFD

**Figure 2:**
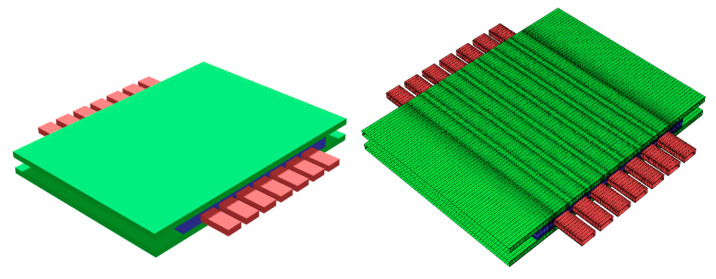
Fluid domain and its discretization.

The thermal-fluid dynamics and performance of the membrane component were investigated by Computational Fluid Dynamic (CFD) approach, using the finite-volume solver ANSYS Fluent 2020R2. The numerical domain (geometry) considered and its spatial discretization (mesh) are shown in Figure 2. The green channels (top and bottom) represent the feed gas domain, the red ones the sweep gas domain, and the blue region is instead representative of the porous support on which the membrane is positioned.

A segregated, pressure-based solver was employed. In particular, the equations needed for the simulation were:

- Mass balance;
- Momentum balance in RANS formulation;
- Transport equation of turbulence quantities (k-omega SST model);
- Energy balance;
- Transport equation of chemical species.

Particular attention was paid to the modeling of the membrane itself. The Arrhenius equation that describes the oxygen permeation through the component was implemented into a sub-routine (user-defined function; UDF) that defines a mass source/sink term in the model. This is an extension of ANSYS Fluent solver itself that, according to local pressure, temperature and chemical species concentrations, models the O_2_ mass source/sink term with the following expression:

$$S_{O2}=\frac{k_{0}}{s}\cdot \exp\left(\frac{-E_{act}}{R\times T}\right)\cdot \;MM_{O2}\cdot \log\left(\left|\frac{P_{O2,\;feed}}{P_{O2,\;sweep}}\right|\right)\cdot \frac{Cell\;area}{Cell\;volume}\;\left[\frac{kg}{m^{3}\;s}\right]$$

where *E_act_* and *k*_0_ are the activation energy and pre-exponential factor, respectively. Both coefficients are determined by permeation test-results.

The boundary conditions for the performed simulations are the following:

- Inlet: imposed velocity, temperature and chemical species concentration;
- Outlet: imposed static pressure;
- Walls: no-slip condition and null mass flux through the boundary, imposed temperature;
- Membrane: permeability modeled with sub-routine (UDF);
- Porous support: porous region with isotropic permeability of 10^−11^ m^2^.

#### 2.3.3. Joining

The assembling of the ceramic component in the metallic case necessarily requires the use of an appropriate sealing material, able to ensure high gas tightness, thermo-chemical and thermo-mechanical compatibility with metallic and ceramic materials, as well as stability in the relevant operating conditions.

In this work, a Ba-based borosilicate glass (GC2) was selected as a joining material for the Inconel 625/LSCF membrane device. Starting products (SiO_2_, CaO, Al_2_O_3_, B_2_O_3_ and BaO) were mixed in proper amount and then melted in a platinum–rhodium crucible into a furnace operating in air for 1 h at 1600 °C. The melt was then cast on a brass plate to obtain the glass, and then it was grounded and sieved to obtain powders (diameter less than 25 µm).

The glass powders were pressed to obtain pellets for the following characterization by hot stage microscopy (HSM), differential thermal analysis (DTA) and X-ray diffraction (XRD), while for the joining process, a slurry was made by mixing the as-prepared powders with a minimal amount of ethyl alcohol. LSCF membranes with dimensions of 15 mm × 15 mm were manually covered on the dense side with the slurry, an Inconel piece was placed on the top of each membrane, and then a thermal treatment, based on results of the thermal characterization, was performed in order to obtain LSCF/GC2/Inconel 625 joints. In particular, the selected joining thermal treatment consists of 2 steps: first, the sample is heated to 950 °C with a heating rate of 2 °C/min and kept at constant temperature for 1 h in order to reach a good sintering degree by viscous flow; then, it is cooled at 850 °C with a dwell time of 2 h to allow the glass to partially crystallize and actually realize the glass–ceramic joint.

Some joined samples (LSCF/GC2/Inconel) were then aged in air for 8 h at 900 °C and then characterized by SEM.

## 3. Results and Discussion

### 3.1. Manufacturing and Scale-Up

The application of scalable manufacturing techniques to the fabrication of membrane components is a key step to enable the diffusion of the membrane separation technology as a valid alternative in industrial production chains where pure oxygen is required. It is also important that the manufacturing route is reliable and reproducible, ensuring a constant quality level of the final components and a low rejection rate.

Therefore, in this study, we focused our attention on the optimization of a manufacturing strategy based on industrially attractive technologies and on the demonstration of its reproducibility. We opted for LSCF as model material, as it is a well-established compound for OTMs that combines sufficiently high oxygen flux and mechanochemical stability [27,28,29,30].

#### 3.1.1. Tape Casting

For the realization of the different layers of the membrane component, we chose tape casting, which is a validated technique to produce planar ceramic structures with large area. The most challenging aspects to obtain green tapes with reproducible quality are the identification of the process parameters (i.e., slurry formulation and drying conditions) and the availability of raw powders with specific properties suitable for such application. We used a commercial powder, produced in large batches, with a specific surface area of 3.5 m^2^/g and a particle size distribution (PSD) in the range 0.1–10 µm with two peaks at 0.5 and 6 µm, which correspond to primary particles and partially sintered agglomerates, respectively, as revealed by SEM analysis (see Figure S4 for PSD and Figure S5 for SEM image).

Due to our previous studies on the optimization of tape casting manufacturing of OTMs [4], we were able to obtain large tapes with constant quality by using this powder as received, without any preliminary powder processing (such as milling or calcination), which would have led to increase time and number of steps in the production chain.

To obtain samples appropriate for the successive manufacturing steps, it is crucial to ensure the thickness uniformity of the tape over its entire length (~100 cm). This can be achieved by carefully controlling the hydrodynamic forces involved in the process, including the hydrostatic pressure of the slurry in the applicator reservoir [31,32]. During the casting process, the slurry height in the reservoir progressively decreases, causing a reduction of the pressure that, in turn, leads to a gradual thinning of the tape. For instance, when casting interlayer tapes without any control of the slurry delivery, we observed a thickness decrease up to 30% from one extremity of the tape to the other. A simple solution that does not introduce additional complexity to the casting set-up is the use of an applicator equipped with two blades, where the additional blade acts as a “metering” system [31] and, if properly positioned, maintains an almost constant slurry amount behind the casting blade during the entire process. By using such a dual blade casting head, we obtained tapes with dimensions of ~100 cm × 40 cm and thickness variations below 5% in the whole area, both for asymmetric and interlayer typologies. A similar homogeneity is acceptable for the following cutting and lamination processes.

#### 3.1.2. Half-Components Fabrication

In order to realize the flow channels required for the sweep gas flow, we cut the interlayer samples by means of punching, a technique widely used for the fabrication of ceramic articles [33].

Based on the results of FEM simulation, we performed punching of seven flow channels with a width of 6.6 mm and a length of 92 mm in the green state, separated among them by stability elements 2.6 mm wide. These dimensions, as well as the interlayer thickness, were decided in order to reach, after sintering, the geometry identified by FEM calculations (see Section 3.3.1), i.e., a channel section of 5 mm × 2 mm and a stability element width of 2 mm. Based on previously published results [3], we used a cutting tool equipped with vertical blades and a deformable rubber matrix to punch green interlayers.

**Figure 3:**
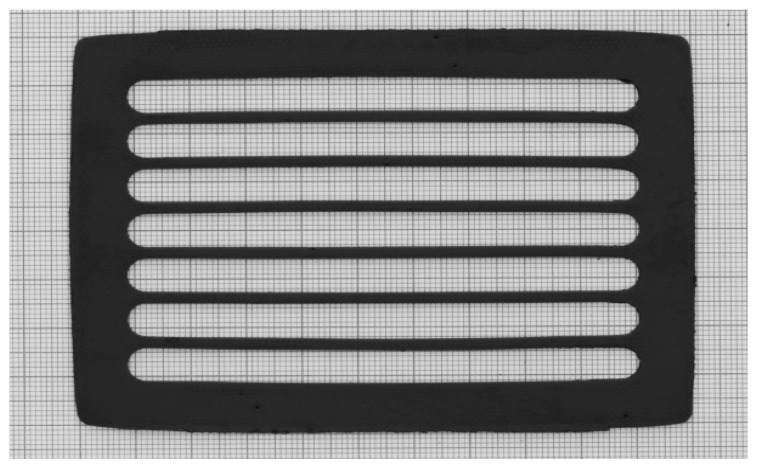
Green interlayer sample (80 mm× 110 mm) after punching out of the flow channels.

We found that the 1.3 mm thick, porous tape can be completely severed by applying a pressure of 14 MPa with a holding time of 10 s, resulting in sharp channel edges without presence of burrs. To prevent a rapid wearing of the tool, as well as the damaging of the thin stability elements, multiple overlapped punching was avoided. We used a high loading rate of 8.75 MPa/s to minimize the blade bending during cutting and to thus achieve a good shape accuracy of the channels cross-sections [3]. Figure 3 shows an interlayer after the punching process.

A half-component is obtained by laminating a punched interlayer with an asymmetric membrane having the same dimension. The two layers, after being stacked one on top of the other, must be heated up to a temperature close to the glass transition temperature of the binder (72–78 °C) in order to enable the viscous flow of the polymer chains in the organic matrix. Subsequently, a pressure must be applied such that the binder phase can flow between the two layers, leading to the joining of the two tapes.

Due to the discontinuous geometry of the interlayers, a careful control of the process is necessary to successfully obtain a laminated sample, preserving the integrity and the stability. We carried out different tests at temperatures between 70 and 80 °C and we identified 70 °C as the best trade-off condition to enable the binder flow without causing an excessive softening of the tape. By running lamination at this temperature, the polymer mobility is sufficient to avoid the formation of cracks at the connection points of the two layers, which are the most critical ones. Conversely, tapes remain sufficiently stiff to retain their geometry, without any sagging of the membrane layer in the underlying channels.

Another crucial parameter is the applied pressure, which must be not too low to prevent delamination, nor too high to avoid excessive deformation of the stability elements. These two requirements can be satisfied by precisely controlling the actual deformation of the stack during the lamination process. Therefore, we performed the lamination tests by controlling the press plates travelling distance as the driving parameter and recording the resulting load that corresponds to the visco-elastic response of the stacked tapes.

**Figure 4:**
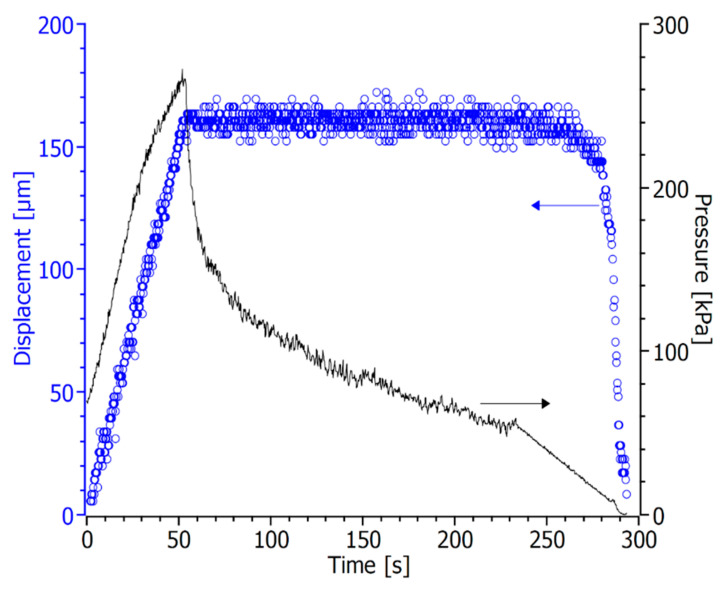
Evolution of sample deformation (○) detected by the extensometer and pressure (−) measured by the load cell during a lamination experiment, assuming *t* = 0 as the instant when contact between the plates and the stack is reached.

Figure 4 gives an insight of the process by depicting the evolution of the plates displacement and the pressure during a typical lamination experiment. Initially, the sample is deformed with a constant rate up to the desired compression value, after which the plates position is maintained for a certain dwell time. During the first part of compression ramp, the pressure increases proportionally to the displacement due to the elastic reaction of the material, while in the final part, the load rising deviates from a linear trend, revealing the onset of a viscous relaxation mechanism. Soon after locking the position of the plates, the pressure detected by the load cell drastically drops, due to the load relaxation through the viscous flow of the binder chains between the two tapes. Such phenomenon persists throughout the dwell time until the pressure reaches a constant value, suggesting that the lamination process is completed. Finally, the residual load, corresponding to the residual elastic response of the stack, gradually releases with a mild rate to prevent delamination.

Laminated samples with good adhesion and excellent geometric stability were obtained by imposing a deformation of 160 µm and a dwell time of 180 s. All the parameters of the optimized process are summarized in Table 2.

Laminated half-components with dimensions of 110 mm × 80 mm were debinded and sintered by means of a thermal treatment that lasts approximatively 140 h, including a slow debinding step to avoid uncontrolled combustion of the organics, followed by a sintering dwell time of 5 h at 1300 °C. The temperature profile is displayed in Figure S6.

**Figure 5:**
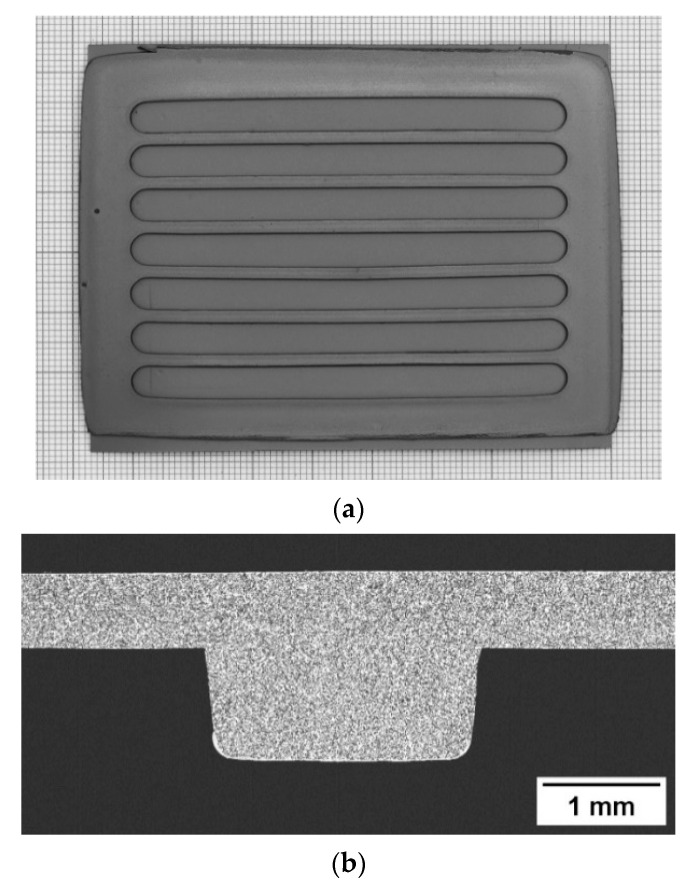
(**a**) Sintered half-component obtained by lamination of an asymmetric membrane and a channeled interlayer. (**b**) SEM cross section of a stability element.

A sintered sample is showed in Figure 5a. The quality of lamination is clear from Figure 5b, where the cross section of a stability element and the adjacent region is depicted. Notably, we applied this lamination process with reproducible results even for different interlayer geometries and/or smaller samples (not showed here), without changing any parameters. This is mainly owed to the direct control of the stack deformation, which can be adjusted to give optimal lamination regardless of the sample properties such as area, layout and stiffness. The only requirements are the proper choice of the lamination temperature, depending on the binder, and a sufficient thickness homogeneity of the starting tapes.

Due to the available equipment, we performed the experiments on 110 mm× 80 mm membranes, but the approach presented in this work can be successfully extended to other samples with different design, and it is particularly interesting for the industrial integration, where high degrees of reproducibility and versatility are required.

Moreover, unlike reported in previous works [3], the whole process can be performed at a constant temperature. This is especially attractive for a serial production chain, since several pieces can be manufactured without consuming time for heating and cooling cycles between consecutive laminations.

#### 3.1.3. Membrane Component Assembling and Finishing

The final processing steps to obtain a complete membrane component suitable to be integrated in the separation module, are the assembling of two half-components, the reworking to proper dimensions, and the sealing of the lateral surfaces.

For the joining of the half-components, we used a garnishing paste prepared with the same LSCF powder used for the membrane manufacturing. Since the half-components were densified at 1300 °C, we sintered the paste at a lower temperature to avoid residual shrinkage of the half-components, which would have damaged the thin membrane layer in contact with the zirconia plate used as support during firing. A sintering step at 1200 °C for 5 h proved to be sufficient to achieve a solid joint.

**Figure 6:**
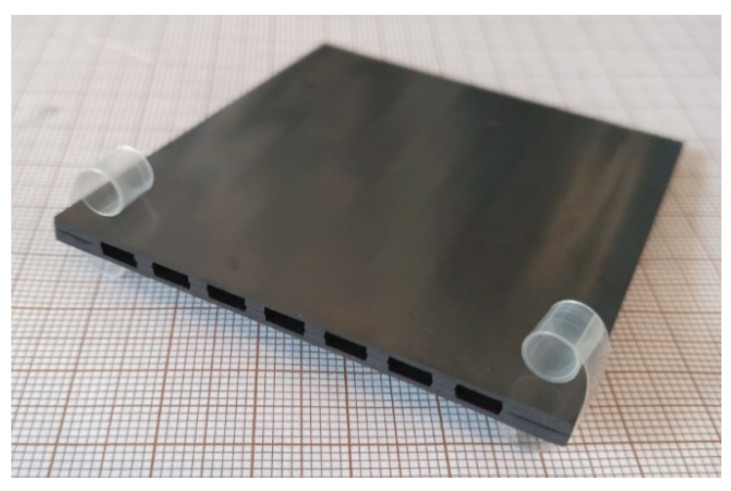
Final membrane component with dimensions of 60 mm× 60 mm.

After garnishing, the lateral regions of the component must be reworked to fit the metal housing and to open the flow channels where sweep gas is supplied during four-end mode operation. Figure 6 shows a final membrane component with dimensions of 60 mm × 60 mm and a total membrane area of 72 cm^2^_._

**Figure 7:**
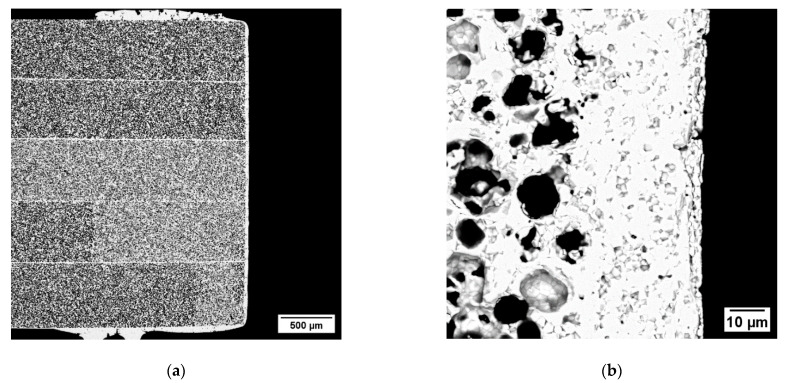
(**a**) Cross-section SEM image of the lateral sealing of a porous sample. (**b**) Detail at a higher magnification of the sealing layer.

In order to assure the gas tightness of the components, the side surfaces parallel to the flow channels must be sealed, as they are mainly composed by porous material. Fully ceramic pastes can be used to this purpose obtaining an adequate sealing, provided that the density in the green state is sufficiently high [34]. We achieved sealing layers with minor porosity (<5%) and no cracks (Figure 7a,b) using the same paste employed for garnishing, due to its high powder concentration (76 wt%). On porous test samples with thickness comparable to the membrane components, we obtained a dense continuous layer by a three-time repeated coating of the edge with a brush, each deposition followed by a sintering at 1200 °C for 5 h. The SEM investigation at higher magnification (Figure 7b) revealed that the sealing layer has a thickness of 30 µm, is well connected to the sample and has no evident defects. The excess paste around the corners (Figure 7a) is caused by the manual procedure and can be easily reduced or avoided by automatizing the application process. Such a sealing layer appears appropriate to ensure the gas tightness of the membrane component edges.

#### 3.1.4. Microstructure of the Membrane Components

**Figure 8:**
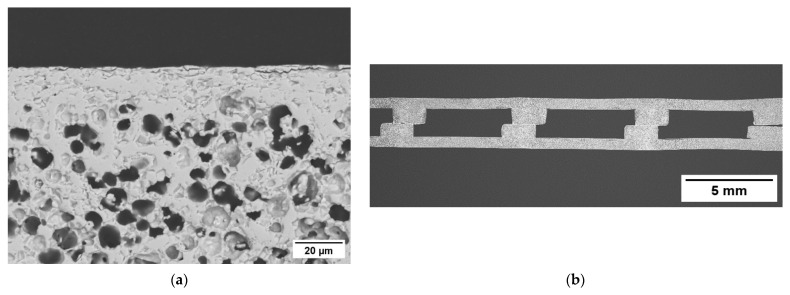
(**a**) Detail of the dense membrane layer and the underlying porous support. (**b**) SEM cross-section image of the membrane component.

Figure 8b shows a cross-sectional view of a final membrane component. The sample has the desired inner structure, with flow channels for the operation with a sweep gas. The channels have a width and height of ~5 and 2 mm, respectively, reproducing quite accurately the architecture of the membrane component designed after FEM analysis (see Section 3.3.1). Based on the thermo-mechanical simulations described in Section 3.3.1, such a structure ensures a remarkable reliability of the component during operation in working conditions, i.e., temperature of 950 °C and pressure difference between the membrane sides of 4 bar. In particular, the predicted failure probability is 2.2 × 10^−6^.

The manufacturing strategy described above is feasible for realizing planar OTM components with such complex configuration, provided that the whole process is carefully controlled. Due to the manual procedures adopted in this study, some aspects can still be optimized, such as the alignment between the two half-components and the homogeneity of the garnishing paste layer. However, all these improvements can be achieved by developing automatized processes when transferring the manufacturing route to a serial production chain. Such inaccuracies do not affect the main target of our work, which is the demonstration of a prototypal complete module appropriate for integration in the industry.

The two asymmetric membranes of the component consist of a thin dense layer with a thickness of ~15 µm (Figure 8a) laying on a porous support whose thickness is ~700 µm. The total porosity of the support calculated by image analysis is 37%, while the open porosity estimated by Archimedes’ method is comparable, indicating that the amount of close porosity is negligible. Such a result is in line with what previously reported for similarly manufactured membranes, where pore network is well interconnected and all pores, in principle, can contribute to the gas transport across the support [3,35]. Conversely, closed porosity would not provide paths for gas transport, while weakening the mechanical strength of the membrane.

### 3.2. Characterization

#### 3.2.1. Permeation Tests

The study of the membrane performance in terms of oxygen flux, defectiveness and stability is interesting for several reasons: first, to compare the results with data reported in the literature and to verify the quality of the manufactured membranes, and moreover, to estimate a semi-empirical law able to describe the oxygen permeation through the asymmetric membrane for the following implementation in a fluid dynamic model of the membrane module.

In order to verify the membrane leakage, room temperature tests were carried out on more than 10 samples and the helium permeance results ranged between 6.0 and 8.0 × 10^−7^ mol/s/m^2^/Pa, confirming the sample density and reproducibility of the manufacturing process.

**Figure 9:**
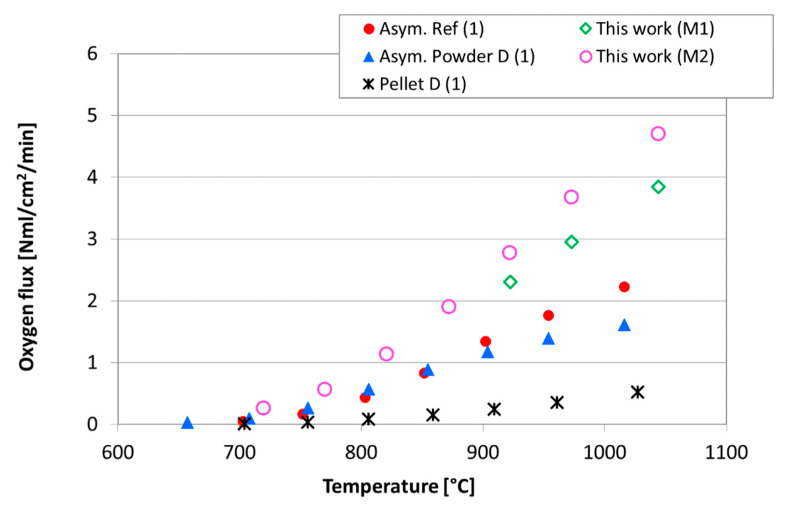
Comparison in terms of oxygen flux as a function of the temperature between samples tested in this work and in [4]. Tests performed by feeding air as process gas.

The oxygen permeation was measured at high temperature and the results collected during tests performed on two asymmetric membranes (named M1 and M2), in the presence of air as process gas, was compared with data reported in [4]. As expected, Figure 9 shows an increase in the oxygen flux with the temperature. At lower temperatures, the permeation values are almost comparable with those reported in literature, while rising the temperature the permeation measured for the samples tested in this work is significantly higher than the reference results [4]. This behavior highlights that at high temperatures, the oxygen ion diffusion through the membrane predominantly controls permeation; hence, the oxygen flux depends on the membrane thickness [36]: the dense layer of samples M1 and M2 is about 18 and 12 μm thick, respectively, while the thicknesses of samples reported in [5] are approximately 20 μm for the asymmetric membranes and 1 mm for the pellet.

Moreover, slight differences can be due to a different experimental set up: tests of this work were performed by feeding air on the membrane side and helium as sweep gas on the support side, with a flow rate of 100 NmL/min, while results reported in [4] were collected by feeding air on the support side, and argon as sweep gas on the membrane layer, with a flow rate of 50 NmL/min.

**Figure 10:**
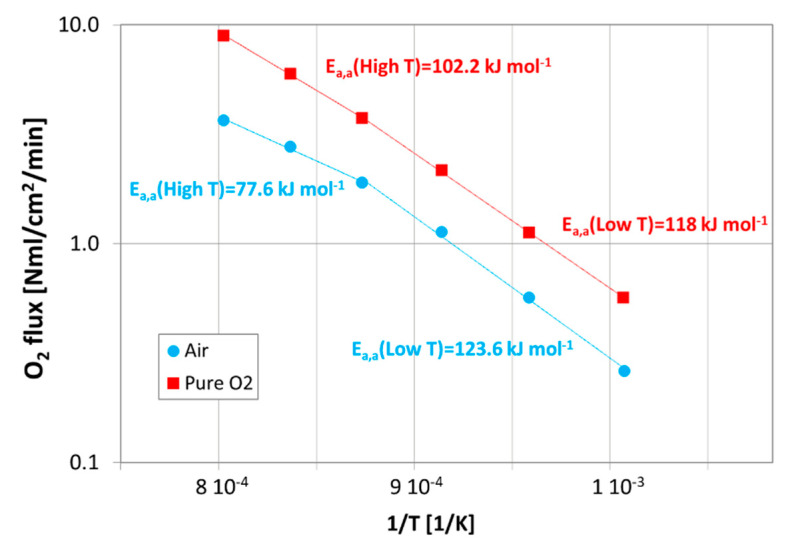
Results in terms of oxygen flux as a function of the inverse of the temperature, collected on the sample M2, both in the presence of air or pure oxygen as process gas, and He as sweep gas (with a flow rate of 100 NmL/min).

The variation of the control mechanism in the permeation process is more evident in Figure 10, which presents the permeation results of the sample M2 measured at high temperature, in the presence of air or pure oxygen as process gas, depicted as an Arrhenius plot, in order to estimate the apparent activation energy (*E*_a,a_) of the permeation process.

Figure 10 shows that a change in the value of the apparent activation energy occurs at about 850 °C, confirming that, according to other authors [36,37], at lower temperatures, the permeation is limited by the surface exchange reactions, while with a rising temperature, the transport mechanism is controlled by the bulk diffusion and the oxygen transport through the porous support. Moreover, this behavior is more evident in the presence of air because these phenomena are strongly affected by the oxygen partial pressure gradient across the membrane. In Table 3, a comparison between apparent activation energy values estimated in this work and reported in [4,37] is presented, showing that our data are in good agreement with literature results.

In addition, for the purpose of fluid dynamic modeling, results collected by varying the sweep flow rate in the range 100–300 NmL/min were further re-elaborated in order to obtain a permeation law describing the permeation process through the whole asymmetric membrane.

**Figure 11:**
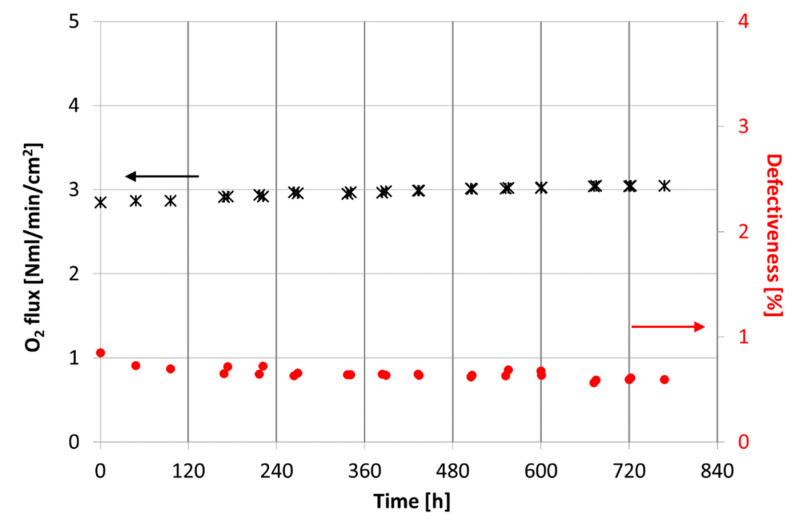
Long-term test results in terms of oxygen flux and defectiveness over time. Tests performed on sample M2 by feeding air as process gas and 100 NmL/min of helium as sweep gas.

After testing the sample M2 at different operating conditions, we verified its stability by carrying out long-term tests and by keeping constant the experimental conditions for nearly 700 h. A test operating temperature of approximately 950 °C was selected according to the OTMs integration studies [12,13]. The results, in terms of oxygen flux and defectiveness over time, are summarized in Figure 11. The oxygen flux plotted in Figure 11 was depurated by the oxygen permeated through the defects, and the membrane defectiveness was estimated as the ratio between the oxygen permeated through the defects and the oxygen permeated through the membrane. More information is provided in the Supplementary Materials. 

A progressive increase in permeation during the membrane operation can be observed and after approximately 700 h at 900 °C, which is about 7% higher than the value measured at the beginning of the long-term tests, while the membrane defectiveness is almost constant and always lower than 1%. This behavior could be due to a progressive formation of cobalt oxide on the membrane layer, caused by the kinetic demixing phenomenon, as already reported by several authors [23,26,28,38,39,40], which occurs during the long-term operation at high temperature and in the presence of an oxygen partial pressure gradient. Since the cobalt oxide, Co_3_O_4_, has catalytic properties in oxidation reactions [41], it can accelerate the oxygen dissociation reaction on the membrane layer and cause a consequent increase in the permeation.

To investigate this hypothesis, at the end of the experimental campaign, the membrane was characterized. By SEM observation, associated with EDS analysis, we did not detect, in the instrument detection limits, any presence of cobalt oxide on the membrane layer, which usually grows on the high oxygen concentration side of the sample [37,39]. This result was also confirmed by XRD measurements performed on the membrane dense layer: the XRD patterns did not show any presence of other crystal phases than perovskite structure of LSCF. Therefore, the oxygen flux evolution over time described above still need to be clarified; possible reasons can be slight microstructural or compositional evolution, not yet detectable, due to the operation time or due to the instruments detection limit.

Aside from the experimental tests in clean conditions, the membrane performance must be studied under realistic gas environments, which can negatively affect the permeation and stability of materials.

OTMs integration studies in industrial processes in a 4-end mode configuration highlighted the high CO_2_-content in the sweep gas stream fed to the membrane module [12,13]. The negative effect of CO_2_ on the permeation of LSCF membranes, already reported in the literature [37,40,42,43,44,45], is due to several phenomena, as the competitive adsorption between CO_2_ and O_2_ on active sites of the membrane surface, the increase in the mass transfer resistance in the porous support or the reaction between the LSCF and the CO_2_ to form carbonates and, consequently, the Sr segregation and the corrosion of the membrane layer. The mentioned phenomena strongly depend on the temperature and on the exposure time and can be reversible when clean conditions are restored or can irreversibly damage the membrane material.

For these reasons, further permeation tests under real gas environments will be performed to better investigate the influence of pollutants on the membrane permeation and on the material thermochemical stability.

#### 3.2.2. Mechanical Characterization

The effectiveness of OTMs is correlated with the pressure difference acting on its opposite faces; the suitability to withstand such pressure difference depends on proper design of the object and intrinsic mechanical properties of the material. Therefore, asymmetric LSCF membranes were characterized in terms of mechanical resistance both at room temperature and at high temperature.

In particular, for the first experiment, we cut 40 samples from an asymmetrical membrane, produced as reported in the previous paragraphs, and successfully tested almost all of them at room temperature. With the aim of verifying any different response to the asymmetry of the membranes, some specimens were tested with the dense side stressed in tension, while others in compression. Subsequently, we carried out a new campaign of high temperature tests at 950 °C on 50 samples, obtained from an asymmetric membrane, again verifying any different response by placing the dense side under compression or tension. As already mentioned for the permeation measurements, the test operating temperature was selected according to the working conditions established in the study of membrane integration in industrial processes [13]. Test results and the associated standard deviations, both for room and high temperature, are summarized in Table 4.

As expected for a ceramic material, data collected both at room temperature and at 950 °C show a certain dispersion. However, since the mechanical tests were performed on several specimens, results can be assumed as descriptive of the LSCF material and used for the following mechanical design of the ceramic component.

**Figure 12:**
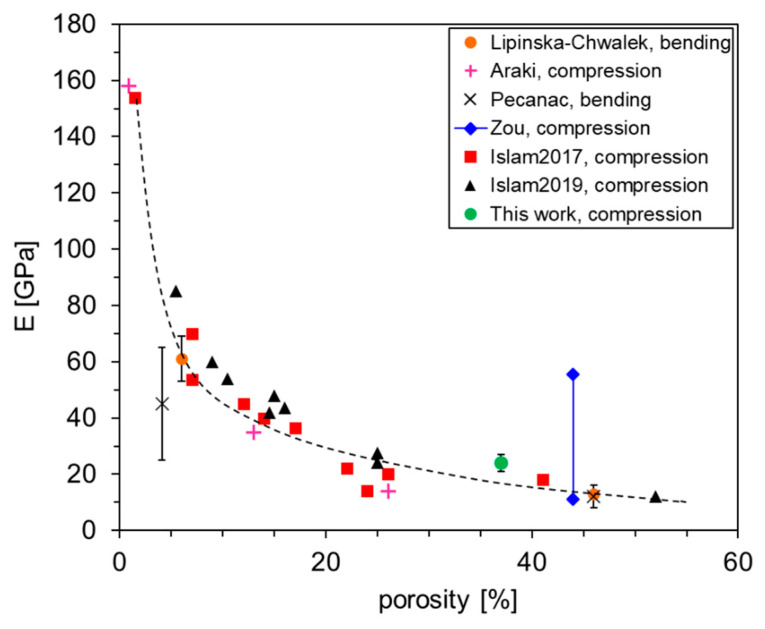
Elastic module measured on the asymmetric LSCF samples of this work compared with results previously reported in literature at RT for porous specimens. The values are grouped with respect to the first author and the characterization technique. The dashed line serves only as an eye guideline.

To the best of our knowledge, this is the first time that a mechanical characterization was performed on membranes with asymmetric structure. Both at room and high temperature, the elastic modulus and the fracture stress do not change, whether the compressive load is applied on the dense or on the porous side. The comparison with results previously given in the literature confirmed that the behavior of the membranes is dominated by the porous support rather than the dense layer, as expected due to the thickness difference between them. This is clear by comparing the value of the elastic module at RT with results reported for LSCF samples with different porosities. Figure 12 displays *E* values measured by various authors using two different techniques, namely, bending test and uniaxial compression test, on specimens with porosity ranging from 0.9 to 52% [46,47,48,49,50,51]. A decreasing trend of the elastic module with increasing porosity can be clearly identified and the value measured in our samples is in good agreement with that expected for a specimen completely composed by a porous layer with a pore fraction of 37%, i.e., the porosity calculated in our specimens.

The increase in the elastic module at 950 °C, with respect to room temperature, matches with what observed by Lipińska-Chwałek et al. [46] in samples with pore fraction of 46%. A similar behavior was ascribed to the phase transition from rombohedral to cubic structure that occurs in LSCF at around 700 °C [27]. It is known that the rombohedral LSCF lattice is characterized by the presence of ferroelastic domains [47,52] that can switch their state when a certain load is applied, causing a relaxation of the stress and a decrease in the module. Moreover, tilting and distortion of the BO_6_ octahedron in ABO_3_ rombohedral perovskite could contribute to relaxing the external load [53]. When raising the temperature above 700 °C, i.e., to temperatures relevant for operation, the crystal structure gradually becomes cubic and, consequently, higher *E* values are measured.

**Figure 13:**
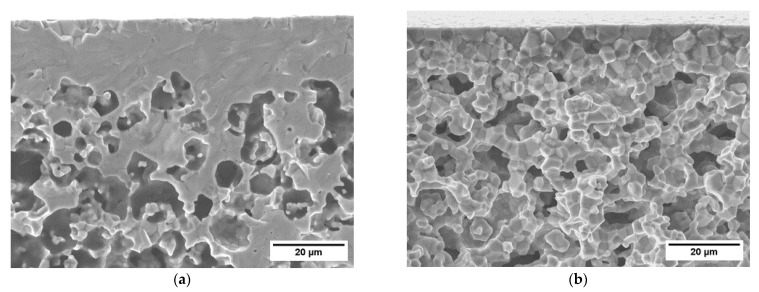
SEM images of fracture surfaces of LSCF specimens after four-point bending tests at (**a**) room temperature (**a**) and (**b**) 950 °C.

The fracture strength increases as well upon heating the membranes up to 950 °C. To better clarify the reasons, we performed fractography by means of SEM. Figure 13 depicts the fracture surface of two samples tested at room temperature and 950 °C, respectively, highlighting that the rupture mechanism is different depending on the temperature. The fracture observed in the RT specimen (Figure 13a) is almost completely transgranular, while at high temperature the fracture occurs in an intergranular mode (Figure 13b). This result agrees with the transition from trans- to intergranular fracture mode pointed out in porous LSCF samples tested by ring-on-ring setup at increasing temperatures of 25, 400 and 800 °C [46]. Such observations indicate that the rise in temperature is accompanied by a change in the limiting factor of the fracture resistance of the material. While at room temperature, the rupture preferentially occurs inside the fragile grains, upon heating the sample the resistance of the grains increases, such that at high temperature the grain boundaries become the weak points through which the cracks can propagate. Accordingly, the overall fracture stress of the specimen increases.

Both in membranes tested at room temperature and at 950 °C, we did not detect any clear fracture origin in the dense membrane layer. This confirms the intuitive hypothesis that, in similar samples, ruptures always originate in the porous support.

#### 3.2.3. Thermal Expansion Characterization

In order to prevent mechanical failures of the membrane module at the operating temperature, it is essential to select, for the metallic housing and the metal-ceramic joining, materials with a thermo-mechanical behavior compatible with the ceramic component. This is a challenge because many oxygen-ion conducting perovskite oxides show peculiar dilatometric properties, determined by the overlapping of the conventional thermal expansion and a chemically-induced strain. The latter is caused by the release of oxygen from crystal lattice, which is accompanied by a reduction of the valence state of the B-site cation and a change in the unit cell volume [54,55]. Chemical expansion becomes particularly important at high temperatures and/or low oxygen partial pressures, conditions that are frequently encountered in many industrial processes.

To investigate the thermo-mechanical properties of the ceramic membranes, in this study, we performed a differential dilatometry characterization on LSCF samples. For ease of manufacturing, we carried out the measurements on dense specimens, but the results can be assumed as representative of asymmetric samples, since thermal expansion of ceramics is not expected to depend on porosity [56].

**Figure 14:**
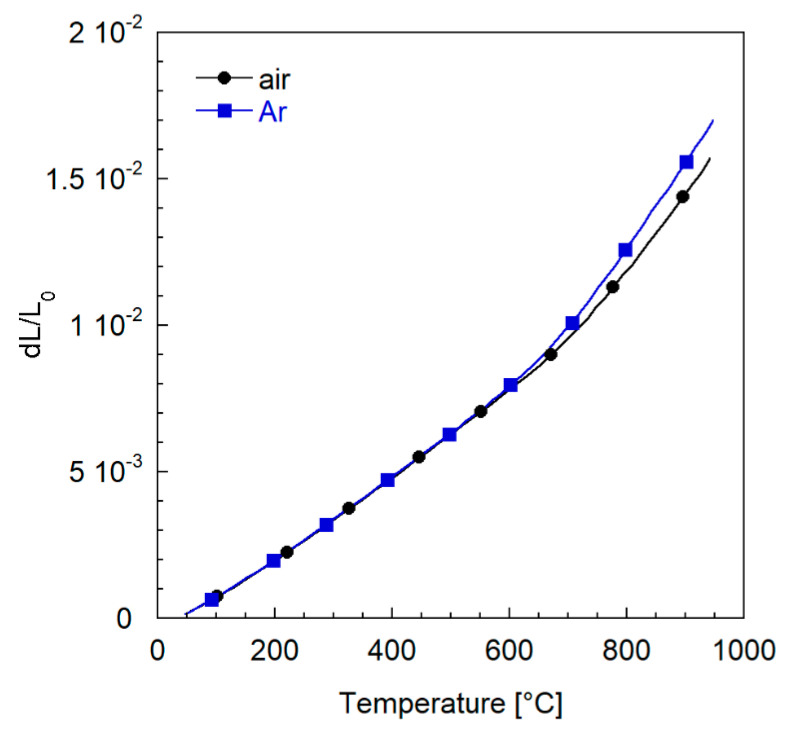
Expansion behavior of LSCF samples in air and Ar atmospheres, from heating curves.

Figure 14 displays the relative expansion d*L*/*L*_0_ recorded by heating the samples from 50 to 950 °C in air and Ar atmosphere. As expected, for temperatures higher than 700 °C, the chemical strain becomes relevant, and both curves deviate from the initial linear trend, the deviation being more pronounced in Ar atmosphere where the oxygen partial pressure is lower.

This behavior reflects in the calculated coefficients of thermal expansion (CTE), reported in Table 5, which shows a significant difference in the high temperature region.

In view of the application of the membranes in the aforementioned operating conditions, i.e., a temperature of 950 °C in air atmosphere, such unusual thermo-mechanical properties of LSCF are a serious issue for the mechanical stability of the module. The main difficulty is that the most common metals used in industrial applications, as well as the possible junction materials, show different behaviors, with considerably lower CTE variations in the whole temperature range. Similar dilatometric properties are hardly compatible with the non-linear trends depicted in Figure 14. Moreover, for *T* > 700 °C, the steep slope of the LSCF curve corresponds to a high value of CTE, which does not fit those of the majority of metals and sealants. Therefore, the identification of module materials suitable to be coupled with the ceramic component without leading to cracks and failures during operation is quite challenging when using LSCF as the membrane material.

### 3.3. Module Design and Joining

#### 3.3.1. Mechanical Design of Ceramic Component and Metallic Case

Outcomes of the mechanical test performed on LSCF samples both at low and high temperature were used to infer strength characteristics of the material and, ultimately, to optimize the shape of a membrane element and to evaluate the probability that it can withstand the requested operating conditions.

**Figure 15:**
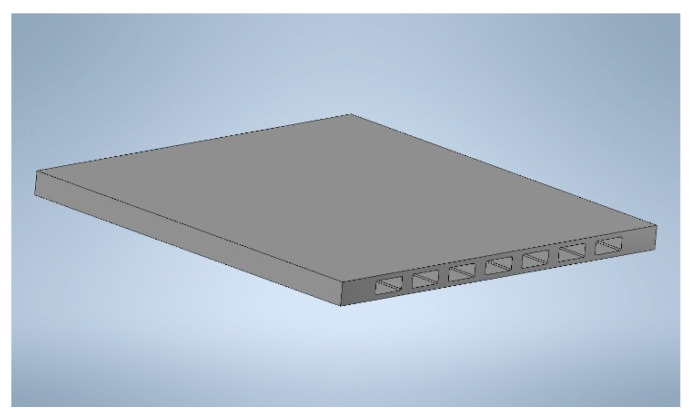
Design of the membrane element with a size of 60 × 60 × ~5 mm^3^.

Three different geometrical configurations were considered for the ceramic component, which differed in the number and size of the flow channels and stability elements, looking for the best compromise to optimize the physical parameters influencing the permeation rate—gases pressure, flow rate, velocity, etc.—and the mechanical resistance of the module itself. Finally, a solution with seven channels 5 × 2 mm^2^ was chosen (see Figure 15).

Mechanical tests performed at high temperature (950 °C) on LSCF ceramic samples led to establishing the following Weibull parameters of the material:

$$m=6.872\;{\left(\sigma_{0}\right)}_{V}=2.9962\;\left[MPa\times m^{0.39718}\right]$$

These values were used to evaluate the failure probability of every cell defined in the FEM model of the ceramic component loaded, with an external pressure 4 bar higher than the internal one and then the failure probability of the whole component, resulting in 2.2 × 10^−6^.

**Figure 16:**
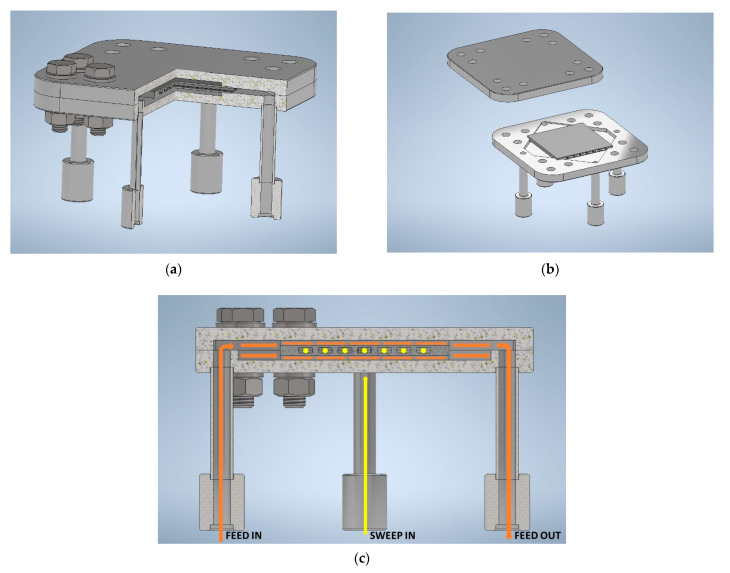
Drawings of the membrane component assembling in the metallic case: (**a**) section view, (**b**) open view, and (**c**) section showing the feed and sweep gas streams during operation.

To operate a ceramic component by keeping the gas streams separated, a metallic case needs to be designed: it consists of two shells forming a housing for the membrane and its sealing material, operating in cross-flow configuration, with air and permeated oxygen (mixed with sweep gas) flowing in mutually perpendicular directions. The assembling of the ceramic membrane component inside its metal case with inlet and outlet tubes for gases is depicted in Figure 16, showing a three quarter partial section view (Figure 16a), an open view (Figure 16b), and the paths of feed and sweep gas streams during operation (Figure 16c).

Gas feeding tubes are welded to metallic case and welding works must be performed before final finishing of coupling surfaces, whose accurate machining is essential. The gas tightness between the two shells of the case relies on the good quality of their machined surfaces.

Housing for the ceramic module in the metal case has to be tailored to the actual ceramic component size, since its final dimensions are prone to some tolerance due to its handcrafted manufacturing and to the limited filling capability of the sealing material, the ceramic component and the metallic case being coupled by means of a glass–ceramic sealing junction.

The extent of possible loss of purity of permeated oxygen due to contamination by air leaks (pressure of the air stream will be higher than that of the sweep gas and permeated oxygen one) will be evaluated with future tests, and its acceptability will be verified.

The main issues are about joining and sealing of the ceramic module and the metallic case—as discussed in the next paragraphs—and the elevated working temperature of the assembly, which causes possible deformation with lose of tightness between different components due to unavailability of sealing devices compatible with these extreme conditions.

#### 3.3.2. CFD

Starting from the FEM results, the fluid dynamic model was developed with the aim of investigating the influence of the module working condition, e.g., the gas velocity and distribution, the pressure gradients that occur inside the module during the operation as well as the local gas concentrations, affecting the membrane permeation efficiency.

In this section, the results of the CFD simulations are reported. The membrane component was tested in three different conditions, varying the velocity of the sweep gas at 950 °C operating temperature. In particular, the operating pressure on the feed and sweep side, the working temperature, as well as the flow rates were inferred according to the results obtained in [12,13].

On a global scale, the variables of interest are:

- Flux of O_2_ permeated through the membrane J(O_2_)_M_;
- Permeation process efficiency, calculated as the ratio between permeated and feed oxygen mass flow rate;
- Oxygen molar fraction at the outlet of sweep channels;
- Oxygen molar fraction at the outlet of feed channels;
- Total pressure drop of the sweep and feed channels.

Table 6 reports the results obtained varying the velocity (and flow rate) in the sweep and feed channels.

**Figure 17:**
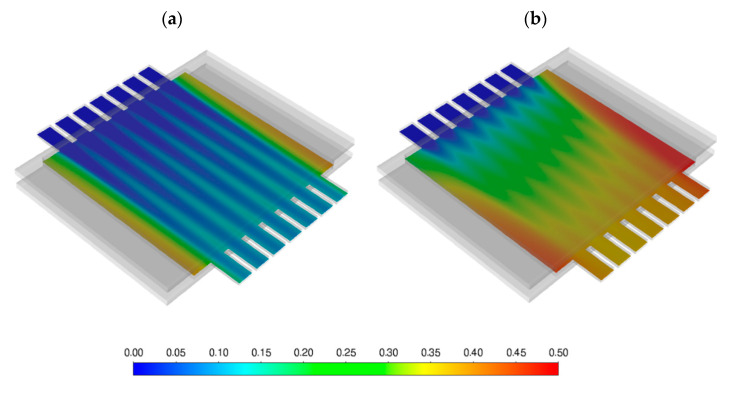
Contours of O_2_ molar fraction, v_sweep_ = 1 m/s (**a**), 0.1 m/s (**b**).

Permeation efficiency increases as velocity in the channels decreases, despite a predictably lower mass flow rate of permeated oxygen. O_2_ molar fraction is sensibly higher than average at the outlet of channels 1 and 7 at higher sweep and feed velocities because they collect the oxygen present on the sides of the porous support, while this does not hold true for the lowest sweep velocity. Diffusion is the predominant mass transport mechanism, and it tends to smear the concentration profiles in the sweep channels. This trend is well described in Figure 17, where the O_2_ molar fraction field on a plane passing at mid height of the porous support is reported, showing the described effect of the sweep velocity (v_sweep_ = 1 m/s in Figure 17a, 0.1 m/s in Figure 17b) on the concentration profiles inside the porous support.

#### 3.3.3. Joining

One of the most critical aspects of the membrane module construction is the identification of an appropriate sealing system, due to the high working temperature of the module. The sealing material has to be able to join two different materials, the ceramic membrane and the metallic case, which exhibit a different behavior at high temperature, by ensuring at the same time a sufficient gas tightness capability and long-term stability [57,58,59].

Glass–ceramic joining materials are widely employed in the energy production sector, due to the feasibility to tailor their properties in terms of wettability, thermal expansion, and thermodynamic stability by choosing the optimal composition. Moreover, glasses can be pressure-less deposited, making the component assembling easier.

The main criteria for the selection of glass-based sealants are the compatibility between the CTE of the sealants and the materials to be joined, as well as the glass transition temperature (T_g_) and the softening temperature (Ts), according to the working temperature of the final device. In particular, the CTE mismatch between each material to be joined needs to be minimized, in order to avoid cracks and fissures during the operation, which can cause irreversible failure of the device.

DTA characterization revealed that the GC2 glass has a T_g_ of 680 °C, a crystallization onset at 833 °C and a crystallization peak at 887 °C. The sintering process, as shown by HSM analysis, occurred in the range 675–778 °C, before the crystallization process. Based on these results, the joining thermal treatment at 950 °C for 1 h and 850 °C for 2 h was selected to obtain the glass–ceramic joint.

The XRD analysis on GC2 glass–ceramic powders detected barium silicate (BaSi_2_O_5_), also known as Sanbornite, as the main crystalline phase after the joining process. An amorphous halo is also present at low 2θ angle, thus suggesting the presence of a residual glassy phase.

**Figure 18:**
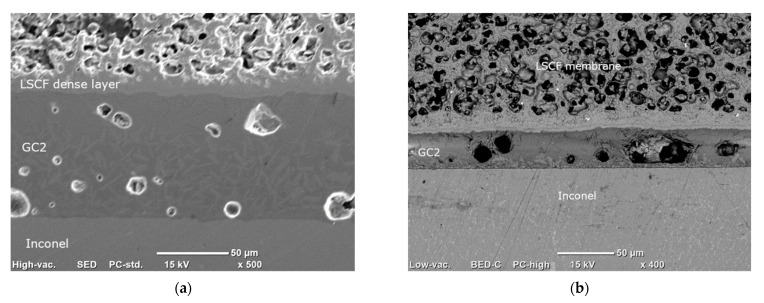
(**a**) SEM cross section of a typical LSCF/GC2 glass–ceramic/Inconel joint and (**b**) LSCF/GC2/Inconel joint after aging 8 h at 900 °C in air. The difference in GC2 thickness between the two samples is due to the manual deposition procedure.

Dilatometric measurements showed that the CTE of the glass–ceramic sealant in air, after the joining treatment, is 11.3 × 10^−6^ K^−1^, almost constant in the whole temperature range 50–900 °C, without any detectable softening peak. Such a value is quite close to that of Inconel 625 alloy (13.1 × 10^−6^ K^−1^) but significantly lower than that of LSCF, mainly due to the chemical strain (see Table 5). Nevertheless, we observed sound interfaces LSCF/GC2 glass–ceramic/Inconel in as-joint specimens. As displayed in the SEM cross-section image reported in Figure 18a, the sealing layer exhibited a good adhesion both to metal and ceramics, resulting in a good joint between glass–ceramic, Inconel and membrane. The presence of isolated pores does not compromise the integrity of the joint. Remarkably, we did not observe any cracks, indicating that GC2 is tolerant to CTE differences.

Pale grey crystals can be observed at the interface between the GC2 and the Inconel layers, while dark grey ones are present in the upper part of the joint, at the interface with the dense LSCF layer. Not only phases with different morphology are involved, but also different concentration and morphology of crystals can be clearly seen. EDS revealed that the crystalline compounds at the interface GC2/LSCF are aluminum-rich, probably aluminum silicates, and at the interface, GC2/Inconel are barium-rich, probably barium silicates.

The SEM cross-section of an aged sample (8 h at 900 °C in static air) is depicted in Figure 18b. It is clear that the adhesion among the layers remained good, without any failures or detachments. We observed a comparable stability in several aged samples having a sealant thickness ranging from 30 to 70 µm. Some isolated cracks and detached regions were observed in only one specimen, where the GC2 layer was ~200 µm thick. This could suggest that an excessive thickness of the joint can be detrimental for the sealant integrity, but some experiments are still in progress to enlighten this point.

Currently, longer stability tests at high temperatures (800–900 °C), coupled with He-leakage and tensile stress measurements, are still ongoing in order to ensure the absence of cracks in the joining layer or diffusion phenomena between the three different materials, which could negatively affect both the gas tightness and the mechanical stability of the assembly.

## 4. Conclusions

In this work, we presented a comprehensive activity aimed at developing a demonstrative planar membrane module for oxygen separation at a high temperature, ready to be integrated into industrial processes. We focused on all the relevant aspects that must be considered in order to achieve a proof of concept device able to validate OTMs technology in real conditions.

We chose LSCF as a membrane model material and optimized the preparation of membrane components with a size of 60 × 60 mm^2^ suitable for the assembling of a module operating both in three- and four-end mode. The component structure, designed combining bending test results and FEM simulations, allows for the supply of a sweep gas through flow channels and ensures a failure probability in operation as low as 2 × 10^−6^. The manufacturing route is reproducible and, due to its flexibility, can be tailored with minor adjustments to produce components with different dimensions and layouts, or by using different ceramic materials.

The membrane characterization in terms of permeation showed results comparable with the literature data. We employed the experimental permeation data to develop a CFD model to assess the influence of the module operating conditions on membrane performance.

The single-membrane component module was designed, paying attention to the joining system between the membrane ceramic material and the metallic case (made of Inconel 625), a well-known critical aspect due to the severe working conditions. To overcome this issue, we used a custom-made glass–ceramic sealant (GC2) that showed a sufficient thermo-mechanical compatibility with both ceramic and metal, resulting in a sound LSCF/GC2/Inconel 625 joint without any failure or detachment among the different materials. Preliminary tests suggested a satisfactory stability of the junction after an aging of about 8 h at 900 °C in air. Tests are ongoing to evaluate the uniformity of the junction on larger (>15 × 15 mm^2^) samples and the influence of prolonged high temperature exposure both on mechanical resistance and gas tightness of the joined assembling.

Finally, the membrane module will be assembled and tested at high temperature and pressure, in order to confirm the success of the module realization and to verify the stability in the identified working conditions.

The approach presented in this study emphasizes that the development of a prototypal module is a multi-disciplinary and complex task. Depending on the process where the module is integrated, mutually influencing design parameters must be tailored accordingly. The results presented in this study highlight an advancement toward the diffusion of OTMs in the industrial sector. However, further research is needed to verify the long-term stable operation of demonstrative devices in a real industrial environment.

## Figures and Tables

**Table 1:** Relevant parameters for the manufacturing of the LSCF layers.

	Casted Layer	Doctor Blade Gap (µm)	Drying Time (h)	Thickness after Drying (µm)
Tape i	Dense	50	1	900
(asymmetric)	Porous	1900	12	
Tape ii	Interlayer	2700	24	1300

**Table 2:** Relevant parameters of the optimized lamination process.

Temperature	70 °C
Plates displacement	160 µm
Displacement rate	3 µm × s^−1^
Dwell time	180 s
Load release time	60 s

**Table 3:** Comparison between apparent activation energy values.

		*E*_a,a_ in Air (kJ/mol)		*E*_a,a_ in Pure O_2_ (kJ/mol)	
Sample	Thickness [µm]	High T	Low T	High T	Low T
This work (M2)	12	77.6	123.6	102.2	118
Asymmetric LSCF [37]	30	72	123	119	146
Asymmetric D [4]	20	44	153	-	-
Asymmetric Ref [4]	20	71	179	-	-

**Table 4:** Results of the symmetric four-point bending tests at room temperature and 950 °C.

Temperature	Dense Membrane Layer in…	# of Tested Specimens	E (GPa)	σ_f_ (MPa)
RT	Compression	18	25 ± 3	36 ± 5
	Tension	17	26 ± 2	34 ± 4
950 °C	Compression	25	59 ± 5	47 ± 2
	Tension	25	57 ± 3	43 ± 1

**Table 5:** CTE derived from heating curves in different atmospheres and temperature regions. Values are expressed in 10^−6^ K^−1^. A comparison with the literature for air atmosphere is also reported [28].

Atmosphere	Air	Ar
pO_2_ (atm)	0.21	10^−5^
CTE 60–700 °C	15.4	14.9
CTE 700–950 °C	26	28.4
CTE 60–950 °C	18	17.7
CTE 60–1000 °C [28]	18.4	-

**Table 6:** Results of the CFD simulations.

T = 950 °C; P_feed_ = 400 kPa; P_sweep_ = 107 kPa			
Eact = 108.57253 kJ/mol			
k0 = 5.5379·10^−5^ mol/s·cm			
s = 0.00122 cm			
**Sweep velocity [m/s]**	**1**	**0.5**	**0.1**
Sweep Flow Rate (m^3^/s) (7 channels)	7·10^−5^	3.5·10^−5^	7·10^−6^
Feed Flow Rate (m^3^/s) (1 channel)	1.017·10^−4^	5.086·10^−5^	1.017·10^−5^
Feed Velocity (m/s)	0.8477	0.4239	0.0848
Permeated O_2_ (kg/s)	3.25·10^−6^	2.79·10^−6^	1.51·10^−6^
Permeation Process Efficiency	8.75%	15.02%	40.56%
	**%O_2_**		
Out 1	14.659	22.292	43.865
Out 2	12.126	18.875	39.994
Out 3	12.072	18.712	38.727
Out 4	12.033	18.614	37.982
Out 5	11.996	18.516	37.327
Out 6	11.967	18.469	37.070
Out 7	14.260	21.389	39.079
Out Feed	17.224	16.450	13.179
	**ΔP_tot_ in-out (Pa)**		
Sweep	5.04	2.59	0.62
Feed	3.61	1.75	0.33

## Data Availability

Not applicable.

## Supplementary Materials

The following supporting information can be downloaded at: https://www.mdpi.com/article/10.3390/membranes12020167/s1, Figure S1: Scheme of the procedure followed for the slurry preparation; Figure S2: (a) Schematization and (b) picture of an asymmetric green tape, composed by a thin dense membrane underlying a thick porous support; Figure S3: Test setup for the four-point bending tests at (a) room temperature and (b) 950 °C; Figure S4: PSD (solid line) and cumulative PSD (dashed line) of the LSCF powder; Figure S5: SEM image of the commercially available LSCF powder used for the manufacturing of the membrane components; Figure S6: Debinding and sintering program of the membrane half-components.
