# Supplementary material for: Development of a Membrane Module Prototype for Oxygen Separation in Industrial Applications
Source: Membranes (Basel). 2022 Jan 30;12(2):167. doi: 10.3390/membranes12020167 (PMC8880189; doi:10.3390/membranes12020167)
Supplement: Supplementary file 1 [file membranes-12-00167-s001.zip › membranes-1558219-supplementary.pdf]

membranes

Article

Development of a membrane module prototype for oxygen separation in industrial applications

Francesca Drago[1,*], Paolo Fedeli[1], Angelo Cavaliere[1], Andrea Cammi[1], Stefano Passoni[2], Riccardo Mereu[2], Stefano De La Pierre[3], Federico Smeacetto[3] and Monica Ferraris[3]

[1] Materials and Generation Technologies Department (TGM), Ricerca sul Sistema Energetico S.p.A. - RSE, 20134 Milan, Italy; francesca.drago@rse-web.it (F.D.); paolo.fedeli@rse-web.it (P.F.); angelo.cavaliere@rse-web.it (A.Cav.); andrea.cammi@rse-web.it (A.Cam.)
[2] Energy Department, Politecnico di Milano, 20156 Milan, Italy; stefano.passoni@polimi.it (S.P.); riccardo.mereu@polimi.it (R.M.)
[3] Department of Applied Science and Technology (DISAT), Politecnico di Torino, 10129 Turin, Italy; stefano.delapierre@polito.it (S.D.); federico.smeacetto@polito.it (F.S.); monica.ferraris@polito.it (M.F.)
* Correspondence: francesca.drago@rse-web.it

Citation: Drago, F.; Fedeli, P.; Cavaliere, A.; Cammi, A.; Passoni, S.; Mereu, R.; De La Pierre, S.; Smeacetto, F.; Ferraris, M. Development of a membrane module prototype for oxygen separation in industrial applications. *Membranes* **2022**, *12*, 167. https://doi.org/10.3390/10.3390/membranes12020167

Academic Editor: Gianluca Di Profio

Received: 30 December 2021
Accepted: 24 January 2022
Published: 30 January 2022

Publisher's Note: MDPI stays neutral with regard to jurisdictional claims in published maps and institutional affiliations.

Copyright: © 2022 by the authors. Submitted for possible open access publication under the terms and conditions of the Creative Commons Attribution (CC BY) license (https://creativecommons.org/licenses/by/4.0/).

Supplementary Material

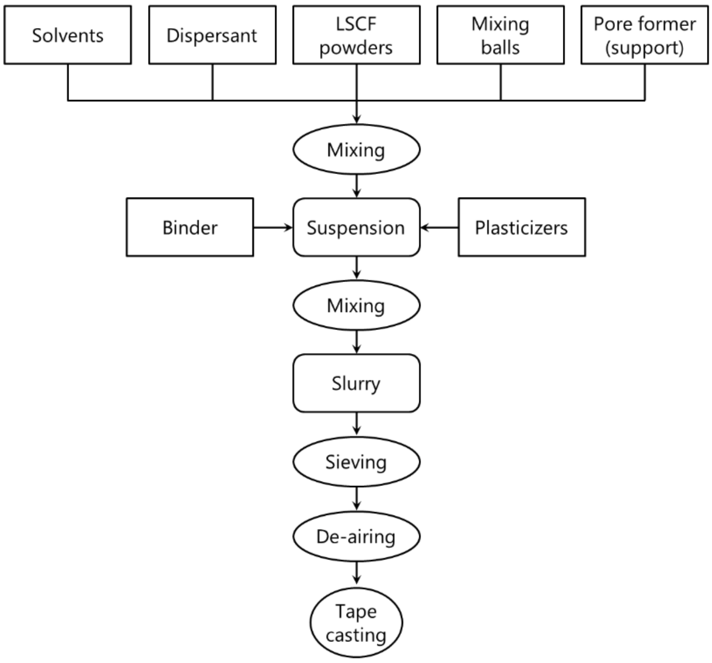

Figure S1. Scheme of the procedure followed for the slurry preparation.

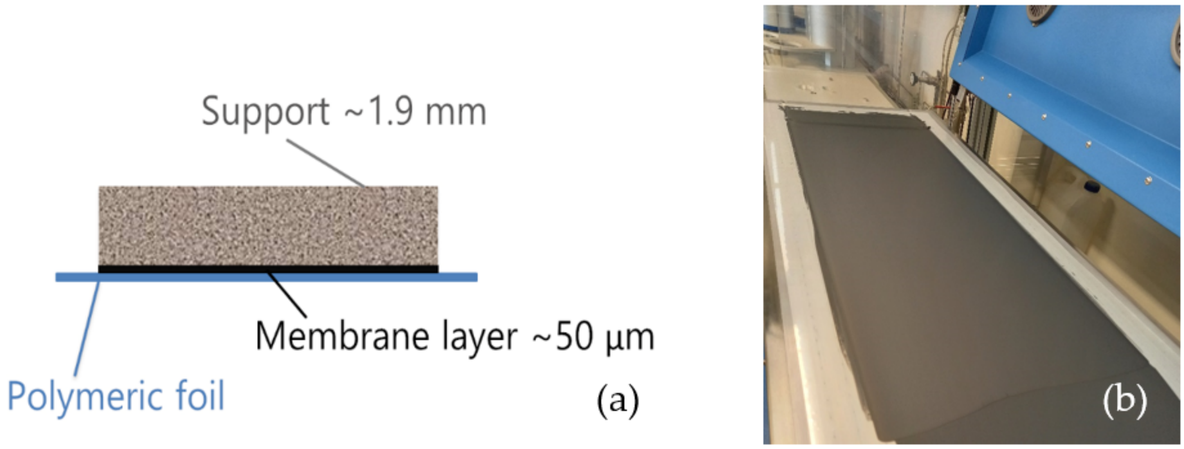

Figure S2. (a) Schematization and (b) picture of an asymmetric green tape, composed by a thin dense membrane underlying a thick porous support. In (a) the doctor blade gaps for casting the different layers are indicated.

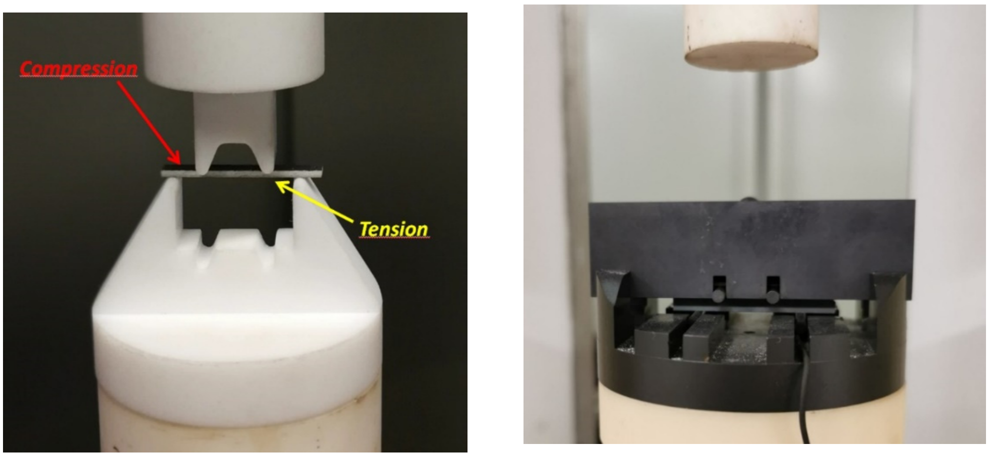

(a) (b)

Figure S3. Test setup for the four-point bending tests at (a) room temperature and (b) 950 °C.

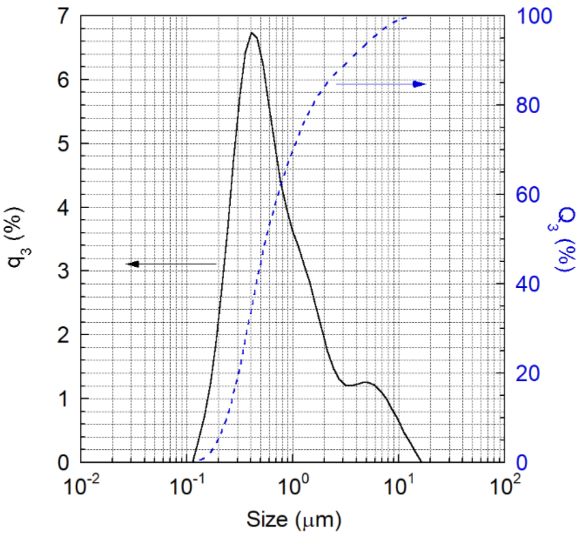

Figure S4: PSD (solid line) and cumulative PSD (dashed line) of the LSCF powder.

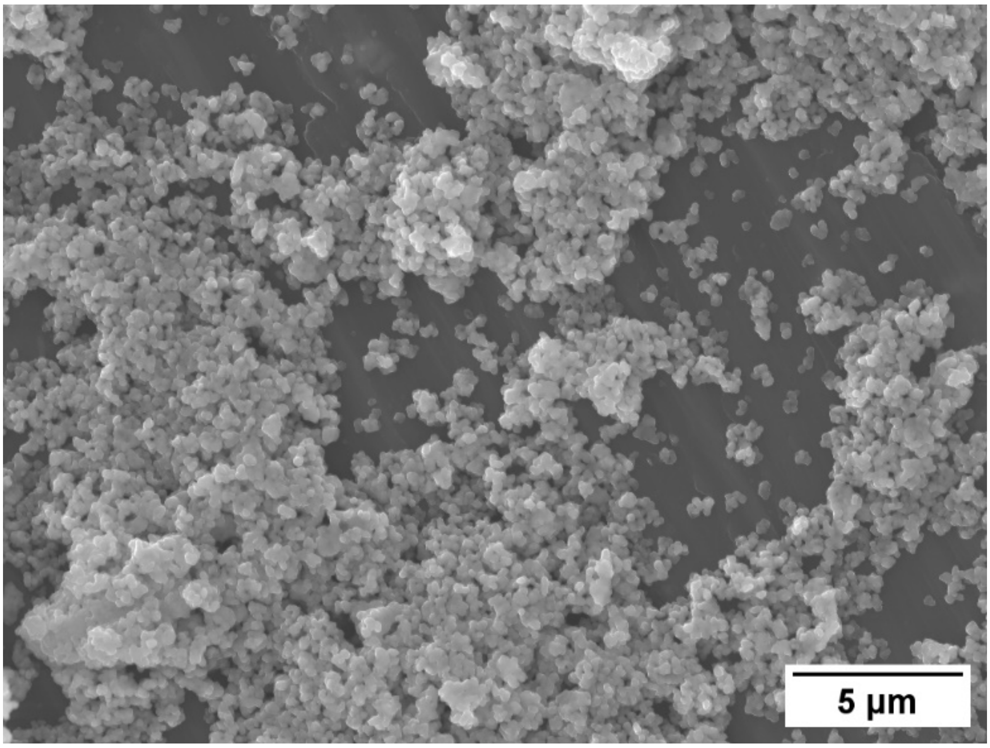

Figure S5. SEM image of the commercially available LSCF powder used for the manufacturing of the membrane components.

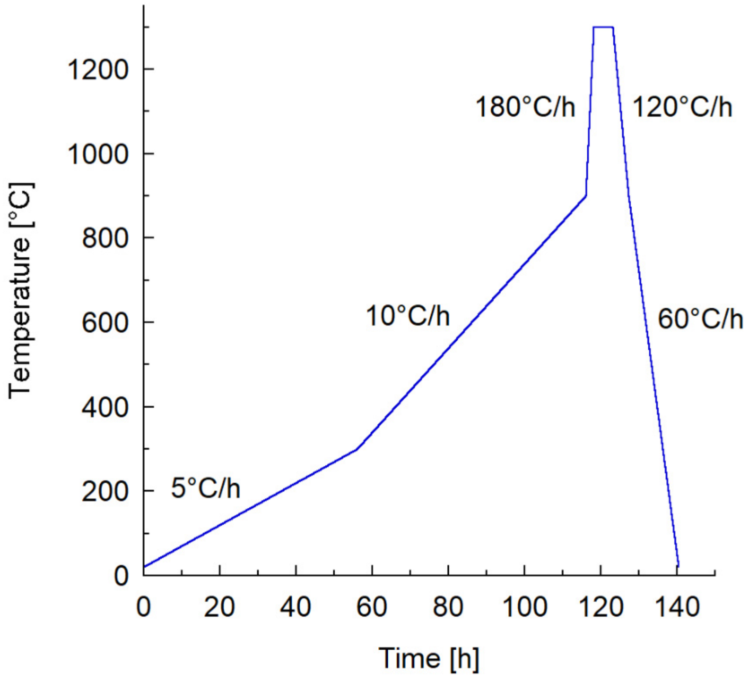

Figure S6. Debinding and sintering program of the membrane half-components.

Oxygen flux estimation

In order to estimate the oxygen flux permeated through the membrane, $J(o_2)_M$, the total oxygen flux, $J(o_2)_{TOT}$, was depurated by the oxygen permeated through the defects, $J(o_2)_D$, according to the following equation:

$$J(o_2)_M = J(o_2)_{TOT} - J(o_2)_D = F(He) * \frac{x(O_2)}{x(He)} - F(N_2)_D * \frac{0.21}{0.79}$$
$$= F(He) * \frac{x(O_2)}{x(He)} - F(He) * \frac{x(N_2)}{x(He)} * \frac{0.21}{0.79} = \frac{F(He)}{x(He)}\left(x(O_2) - x(N_2) * \frac{0.21}{0.79}\right)$$

Where $x(He)$, $x(O_2)$ and $x(N_2)$ are the helium, oxygen and nitrogen concentration, respectively, obtained by microGC measurements, $F(N_2)_D$ is the estimated nitrogen flux which permeates through the defects, $F(He)$ is the sweep gas flow rate, controlled by a mass flow controller, while $\frac{0.21}{0.79}$ is the ratio between oxygen and nitrogen concentration in the feed gas stream.
